# Editorial: An interdisciplinary perspective on resilience - A special section in *Neuroscience Applied*

**DOI:** 10.1016/j.nsa.2024.104044

**Published:** 2024-02-15

**Authors:** Stella D. Voulgaropoulou, Thomaz F.S. Bastiaanssen, Nuno D. Alves, Aurelia Viglione, Joeri Bordes, Benjamin Jurek, Pasquale Paribello, Milou S.C. Sep

**Affiliations:** Department of Psychiatry and Neuropsychology, School for Mental Health and Neuroscience (MHeNs), Maastricht University, Maastricht, the Netherlands; Department of Anatomy & Neuroscience, APC Microbiome Ireland, University College Cork, Ireland; Department of Psychiatry, Amsterdam University Medical Centers Location Vrije Universiteit Amsterdam, the Netherlands; Life and Health Sciences Research Institute (ICVS), School of Medicine, University of Minho, 4710-057, Braga, Portugal; ICVS/3B's—PT Government Associate Laboratory, 4710-057, Braga, Guimarães, Portugal; Istituto Superiore di Sanità, Center for Behavioral Sciences and Mental Health, Rome, Italy; Neurocentre Magendie, INSERM 1215, Université Bordeaux, Bordeaux, France; Neurobiology of Stress Resilience Group, Max Planck Institute of Psychiatry, Munich, Germany; Section of Psychiatry, Department of Medical Sciences and Public Health, University of Cagliari, Cagliari, Italy; Department of Psychiatry, Amsterdam University Medical Centers Location Vrije Universiteit Amsterdam, the Netherlands; GGZ InGeest Mental Health Care, Amsterdam, the Netherlands; Amsterdam Neuroscience, Mood, Anxiety, Psychosis, Sleep & Stress Program, Amsterdam, the Netherlands; Amsterdam Public Health, Mental Health Program, Amsterdam, the Netherlands

Welcome to this Special Section in *Neuroscience Applied*, offering an interdisciplinary perspective on Resilience. Resilience is a complex phenomenon that is studied by a wide array of scientific disciplines, including psychology, psychiatry, neuroscience, immunology and biochemistry. This special section is put together by the Resilience Network of the European College of Neuropsychopharmacology (ECNP) (ECNP, 2024). The aim of this interdisciplinary network of resilience researchers is to change the current approach of performing resilience studies by pooling knowledge, consolidating efforts, and fostering data-sharing to accelerate research identifying who is resilient to stress and which factors contribute to an optimal stress resilience.

In this editorial, the Early Career (EC) Team of the ECNP Resilience Network (Fig. 1) highlights how this Special Section reflects the network's mission. The interdisciplinary EC team developed three main pillars to translate the Network's ambitions into practice.

(1) **Transversal, replicable, and open research**: Establish universal and applicable experiments, translational models, and interventions to study resilience. Share and discuss novel research concepts, ideas, and technologies within the field.
(2) **Integrative framework**: Promote and implement a holistic approach to the wide, complex, and diverse field of resilience research, moving from an individual-centered perspective of resilience to an integrative one.
(3) **Updated and unifying communication, within and outside of the scientific community:** Renew the most commonly used concepts and terminology in the field. Disseminate the most recent advances through research articles, podcasts, webinars, etc.

**Fig. 1:**
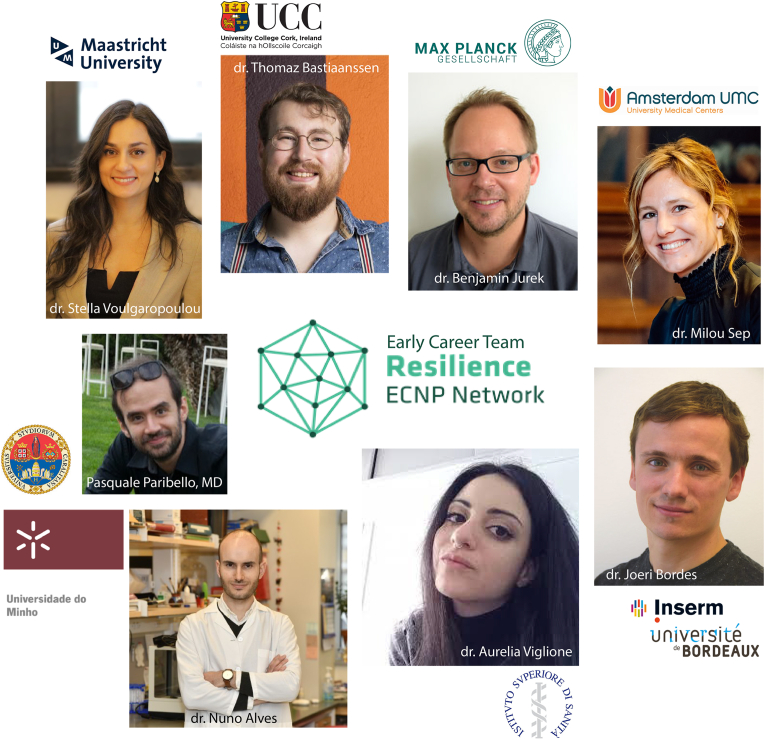
The early career team of the resilience network from the european college of neuropsychopharmacology (ECNP).

The works in this special section reflect these three pillars of interdisciplinary resilience research.

**Transversal, replicable, and open research**: Sep et al. highlight the STRESS-EU database (www.stressdatabase.eu), which contains individual participant data (IPD) of human acute stress studies (n = 6576) from 16 European research groups. It includes a variety of stress-induction paradigms and stress-related outcomes in healthy and patient populations and data is available to the broader community of stress and resilience researchers. Albayrak and de Fatima da Silva Vaz et al. review various (translational) stress models suitable for assessing stress resilience, highlighting key factors such as the temporal aspect of stress exposure and the sex of the subject that should be considered for future experiments.

**Integrative framework:**Paribello et al. performed a quasi-systematic review of studies describing some of the most promising clinical and preclinical models of biomarkers for the dyadic interaction of stress-resilience, focusing on inflammation, the endocannabinoid system and the hypothalamus pituitary adrenal axis. They conclude that methodological and translational issues hamper the clinical utility of stress-resilience biomarkers. van Hooijdonk and Voulgaropoulou et al., provide a systematic review examining how genes encoding the norepinephrine transporter and receptors may impact specific characteristics of resilience related to mental health, such as cognitive processes, personality traits, and emotional memory in a transdiagnostic manner.

**Updated and unifying communication:**Pasteuning and Gathier et al. present a novel multilevel dynamic framework to tackle the high levels of heterogeneity in the literature on resilience after childhood adversity. Their framework explicitly integrates the contextual diversity of childhood adversity, the heterogeneity in resilience operationalizations, and the time dynamics of resilience after childhood adversity to increase transparency in future work and facilitate the synthesis of research findings.

## Towards a more resilient future

Together, the works in this special section reflect the unifying approach to interdisciplinary resilience research that will lay a solid foundation for future studies, providing a common framework and understanding that enhances the coherence and impact of resilience research. The purpose of this special issue is two-fold. First, it underlines the multidisciplinary nature of resilience research and introduces numerous parallel approaches to study resilience. Second, it highlights research articles already leveraging multidisciplinary approaches to help shape future directions of the resilience field. We are excited to share our vision for the future of resilience research, which entails a multidisciplinary, integrative approach, crucial to understand the mechanisms underlying resilience. It also requires a diverse group of senior *and* early career scientists working together. We hope our readers enjoy this special issue and that it may encourage a more holistic, multidisciplinary view of resilience.

## Declaration of competing interest

The authors declare that they have no known competing financial interests or personal relationships that could have appeared to influence the work reported in this paper.
